# Dextran sulfate-based MMP-2 enzyme-sensitive SR-A receptor targeting nanomicelles for the treatment of rheumatoid arthritis

**DOI:** 10.1080/10717544.2022.2032482

**Published:** 2022-02-04

**Authors:** Caiwei Yu, Hui Liu, Chunjing Guo, Qiang Chen, Yanguo Su, Huimin Guo, Xiaoya Hou, Feng Zhao, Huaying Fan, Hui Xu, Yan Zhao, Xiaofeng Mu, Guohua Wang, Haiyu Xu, Daquan Chen

**Affiliations:** a Key Laboratory of Molecular Pharmacology and Drug Evaluation, Ministry of Education, Collaborative Innovation Center of Advanced Drug Delivery System and Biotech Drugs in Universities of Shandong, School of Pharmacy, Yantai University, Yantai, P. R. China; b Department of Pharmacy, The Affiliated Yantai Yuhuangding Hospital of Qingdao University, Yantai, Shandong, P. R. China; c College of Marine Life Science, Ocean University of China, Qingdao, P. R. China; d Institute of Chinese Materia Medica, China Academy of Chinese Medical Sciences, Beijing, China

**Keywords:** Rheumatoid arthritis, celastrol, dextran sulfate, MMP-2 response, targeting drug delivery system

## Abstract

Rheumatoid arthritis (RA) is an ordinarily occurring autoimmune disease with systemic inflammatory. Targeted drug delivery systems have many successful applications in the treatment of rheumatoid arthritis. In order to develop nanoparticles for targeted delivery of Celastrol (Cel) to rheumatoid arthritis and specific drug release, the dextran sulfate (DS) was modified as the targeting molecular by binding to the scavenger receptor of macrophage. The dextran-sulfate-PVGLIG-celastrol (DS-PVGLIG-Cel), named DPC, amphiphilic polymeric prodrug was synthesized and characterized. The resulting DPC@Cel micelles had the average size of 189.9 nm. Moreover, the micelles had ultrahigh entrapment efficiency (about 44.04%) and zeta potential of −11.91 mV. In the *in vitro* release study, due to the excessive production of matrix metalloproteinase-2 (MMP-2) at the inflammatory joint, the MMP-2 reactive peptide was used to crack in the inflammatory microenvironment to accelerate the release of Cel. The results have shown that the nanoparticles can effectively deliver Cel to activated macrophages and significantly improve the bioavailability. *In vivo* experiments showed that DPC@Cel have better anti-rheumatoid arthritis effects and lower systemic toxicity than free Cel. This study provided a new therapeutic strategy for the treatment of RA.

## 1. Introduction

Rheumatoid arthritis was the most common autoimmunity inflammatory arthropathy that causes progressive joint inflammation associated to bone, cartilage erosions, and comorbidity (Cush 2021). RA represented a major global public health challenge, 0.3–1% of the general population in the world suffered from it (Boutet et al. 2021). At present, there were many commonly used drugs for treating rheumatoid arthritis in the market. However, long-term use of these drugs could lead to severe side effects, including systemic diseases such as liver and kidney, and dysfunction of gastrointestinal (Yang et al. 2017). The rapidly developing self-assembled nanoparticles were used as effective targeting carriers to improve the stability, biological safety, and effectiveness of hydrophobic drugs, and are widely used in anti-cancer, anti-inflammatory, and other diseases (Cheng et al. 2019；Liu et al. 2019; Fang et al. 2020; Hou et al. 2020).

Celastrol was a pentacyclic triterpene compound extracted from Tripterygium wilfordii, belonging to the quinone methide family (Bao and Dai 2011; Chen et al. 2018；Luo et al. 2019). Celastrol has shown the ability to inhibit the development of rheumatoid arthritis, obesity and metabolic dysfunction and the proliferation of different cancer cells (Kashyap et al. 2018; Tang et al. 2018; Zeng et al. 2018). Celastrol’s shortcomings were poor water solubility and low bioavailability, which greatly limited its effect. To resolve this drawback, amphiphilic polymer had been adopted. Macrophages played a very central pathogenic role in the development of RA (Sun et al. 2017; Keewan and Naser 2020). Fibroblast-like synovial cells contributed to the pathological changes in the rheumatoid synovium and the propagation of inflammation. Accumulating evidence had implicated celastrol inhibits RA-FLS proliferation by inducing apoptosis in vitro, DNA damage and cell cycle arrest (Tice et al. 2000; Xu et al. 2013).

Matrix metalloproteinases (MMPs), were members of the group of zinc-dependent endopeptidases. A recent study showed that the MMP-2 enzyme is excessively secreted in the joints of patients with rheumatoid arthritis and plays an important role in inflammation and immunity. (Giannelli et al. 2004). Xue et al. (2014), demonstrated that MMP-2 inhibited degradation of cartilage and RA-synovial fibroblast mediated inflammation. Therefore, in our study, we introduced the MMP-2 sensitive peptide (PVGLIG peptide) to achieve specific release in the inflamed joints. The development of a nanocarrier with MMP-2 enzyme response to treat RA was a new strategy and provided a new idea for the treatment of RA in the future.

Activated macrophages were abundant in the joints of patients with rheumatoid arthritis, which ultimately leads to inflammation and joint destruction (McInnes and Schett 2017 ; Yang et al. 2017; Smolen et al. 2018). Consequently, the activated macrophages made a suitable candidate for targeted therapy of RA. Dextran sulfate (DS) was a widely used biological material. It was a negatively charged hydrophilic polysaccharide with significant biocompatibility and biodegradability(Pardeshi and Belgamwar 2017; Yucel Falco et al. 2017). DS was a representative ligand for activated macrophage scavenger receptor class A (SR-A) (Kim et al. 2013; Heo et al. 2017). Type A scavenger receptor (SR-A), also called CD204, is mainly expressed on activated macrophages (Hashizume and Mihara 2012; Yu et al. 2020). Using dextran sulfate as the hydrophilic core of an amphiphilic polymer could enable nanoparticles to actively target activated macrophage. Therefore, this strategy became an ideal drug carrier for the treatment of RA.

**Figure 1.**
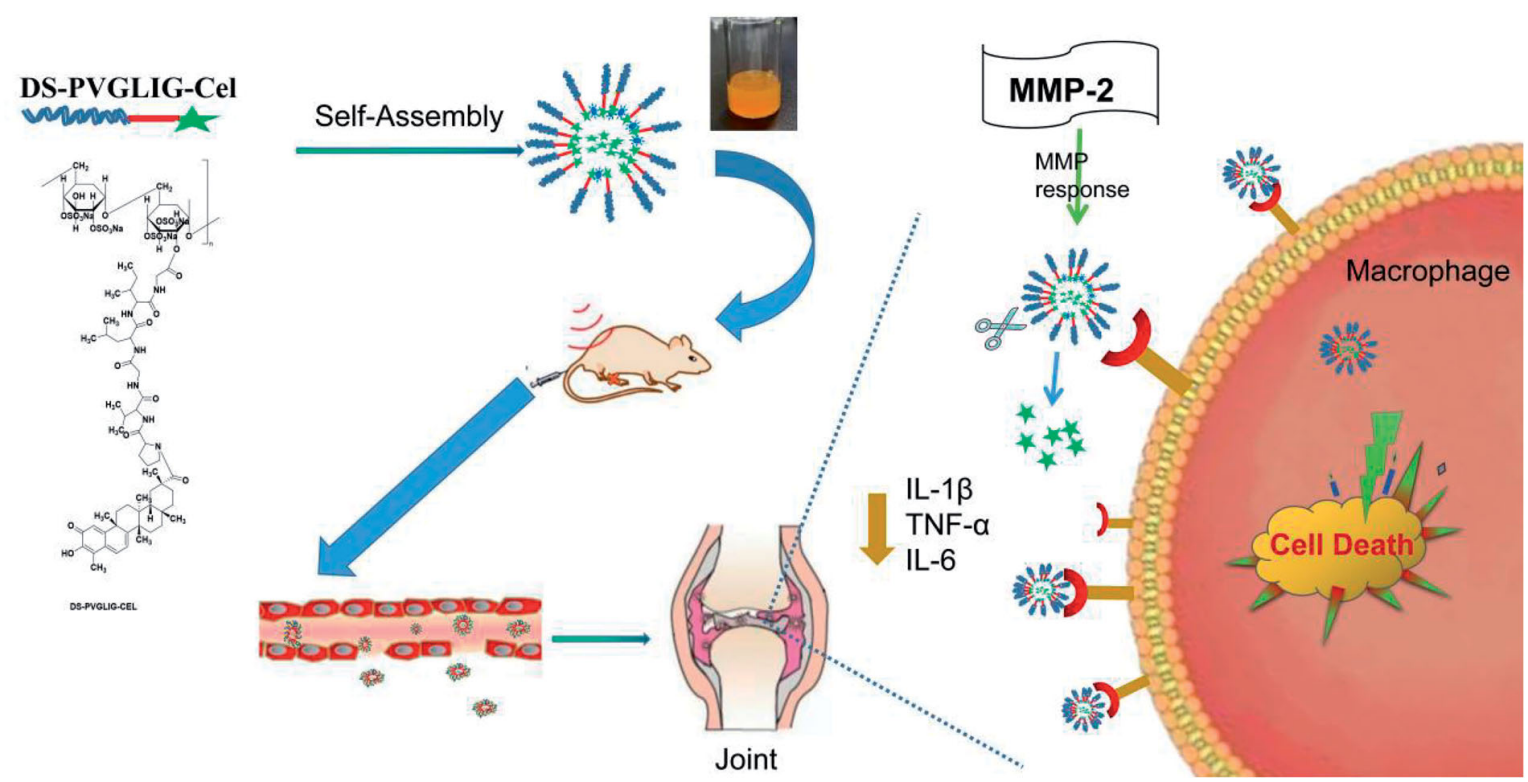
Design and schematic illustration of DPC@Cel.

Based on this, we designed a polymer prodrug with celastrol as a hydrophobic core, in which dextran sulfate not only served as the hydrophilic block, but also targeted the SR-A on the surface of macrophages. βAla-Pro-Val-Gly-Leu-Ile-Gly-βAla-Cys (PVGLIG) peptide as an MMP-2 responsive linker was connected celastrol and DS. Based on the high level of MMP-2 enzyme in inflamed joints, we hypothesized that PVGLIG could be hydrolyzed and broken by MMP-2 enzyme in the microenvironment. Celastrol was exposed to increase the drug concentration in the tissue environment, and achieve better therapeutic effects (Figure 1).

## 2. Materials and methods

### 2.1. Materials

DS (5000 Da) was received from OLBASE reagent net, PVGLIG was purchased from Nanjing Laiang biological Co., Ltd. Celastrol was purchased from Nanjing Chunqiu biological Co., Ltd. 1-(3-Dimethylaminopropyl)-3-ethylcarbodiimide hydrochloride(EDC), N-Hydroxy succinimide(NHS) and 4-Dimethylaminopyridine(DMAP) were obtained from OLBASE reagent net. MMP-2 was purchased from Sigma-aldrich. Raw 264.7 (Mouse mononuclear macrophage leukemia cells) was got from Shanghai Guandao Biological Engineering Co. Fibroblast-like synoviocytes-rheumatoid arthritis cells (RA-FLS) was purchased from Shanghai Guandao Biological Engineering Co., Ltd. MTT was got from OLBASE reagent net.

### 2.2. Methods

#### 2.2.1. Synthesis of DS-PVGLIG-Cel

Based on hydrophilic dextran sulfate, amphiphilic carrier material DPC was synthesized.

##### 2.2.1.1. Synthesis of PVGLIG-Cel

Step 1:Cel (18.4 mg, 0.1 mM), EDC (15.2 mg, 0.15 mM), and NHS (9.2 mg, 0.15 mM) were mixed and dissolved in 1 mL of DMSO to activate at 35 °C for 3 h. PVGLIG (22.16 mg, 0.1 mM) was then added to the reaction flask. The reaction was carried out at 35 °C for 36 h.

##### 2.2.1.2. Synthesis of DS-PVGLIG-Cel

**Figure 2.**
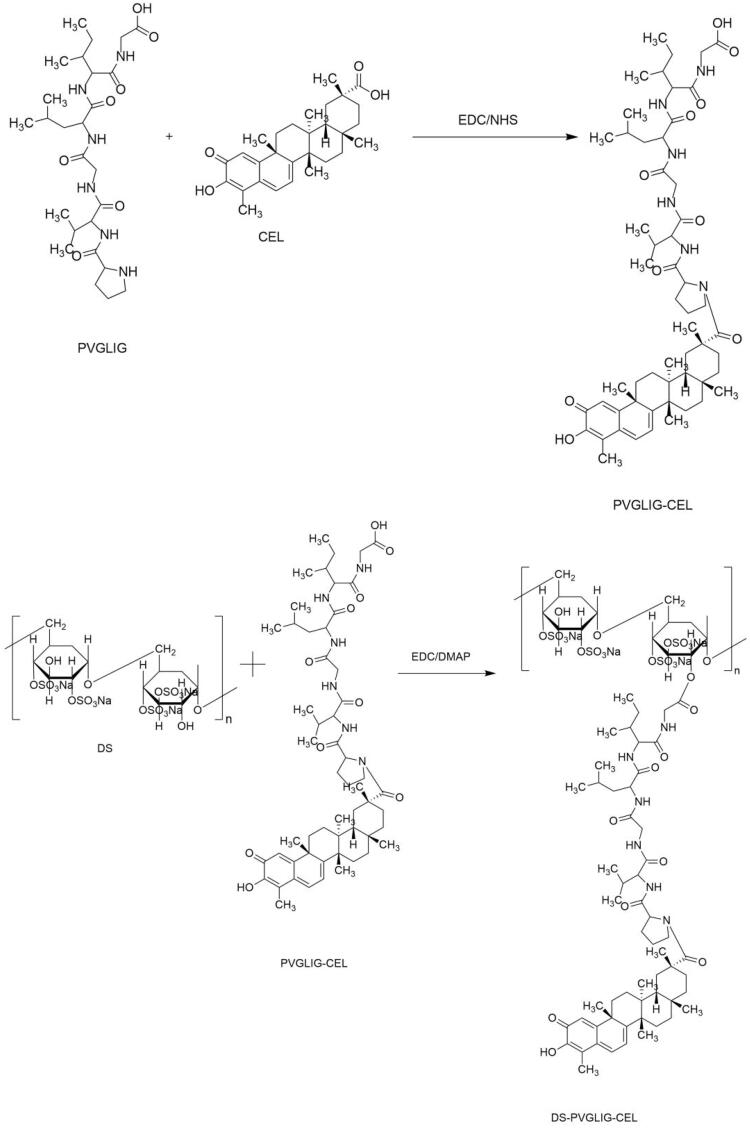
(1), (2) Synthesis and characterization of DPC.

Step 1:EDC (15.2 mg, 0.15 mM), DMAP (17.3 mg, 0.15 mM) and DS (38.88 mg, 0.18 mM) were added in 2 mL of DMSO. Then, continue the reaction at 35 °C for 48 hours. Finally, in deionized water, dialyze the mixed solution with a dialysis bag (3500 Da, MWCO) for 24 h. And then, the solution was vacuum freeze-dried. The final product (DS-PVGLIG-Cel, DPC) (Figure 2) was finally obtained-an yellow floc.

#### 2.2.2. Characterization of DPC

The structure of DPC was characterized by 1H-NMR spectroscopy and FTIR. Using a mixture of DMSO-D6 and D_2_O as a solvent, 6 mg of DPC was dissolved in it. 1H-NMR of DPC, PVGLIG-Cel, DS, PVGLIG and Cel were measured.

#### 2.2.3. Preparation of Cel-loaded DPC micelles (DPC@cel)

We used the dialysis method to load the celastrol package into the DPC. Specifically, through prescription optimization, dissolve 10 mg DPC in a mixed solvent of DMAP and DMF(DMAP: DMF = 1: 0.8), and dissolve 1 mg Cel in 1 ml DMAP. The next step was stirring and mixing the two with a magnetic stirrer. Use a dialysis bag (MWCO 3500 Da) to dialyze the mixed solution in deionized water for 24 hours, and replaced the deionized water every 2 hours.

#### 2.2.4. Characterization of DPC@cel

##### 2.2.4.1. Particle size, zeta potential, morphology of micelles under TEM (TEM, H-600; Hitachi, Tokyo, Japan)

The transmission electron microscope was used to observe the morphology of Cel-loaded DPC micelles clearly. The particle size and polydispersity index (PDI) of DPC@Cel were determined by using Delsa Nano C Particle Analyzer. Zeta potential was also determined by using Delsa Nano C Particle Analyzer.

We evaluated the stability of DPC@Cel by measuring the change in particle size over time. We put DPC@Cel in the PBS buffer solution with PH = 7.4 and containing 20% FBS. We simulated the serum microenvironment and added 1.5/mL of MMP-2 enzyme to the release medium. And we stored them in a refrigerator at a constant temperature of 4 °C. We take it out at 12, 24, 48, 72 h, then measure the particle size.

##### 2.2.4.2. Encapsulation efficiency (EE) and drug loading capacity (DL) of DPC@Cel


(1) EE%= (weight of Cel encapsulatin micelles)/(total weight of feeding Cel)× 100%

(2) DL% = (mass of Cel encapsulted in micelles)/(Total Mass of the micelles) × 100%

High Performance Liquid Chromatography(HPLC, Agilent 1260GB12C) was also selected to test the encapsulation efficiency (EE%) and drug loading (DL%) of DPC@Cel. The methods (Equations (1) and (2)) for calculating the EE% and DL% of DPC@Cel were listed above. First, we took the DPC@Cel 1 ml after the 0.88 μm microporous filter membrane, and diluted it to 10 ml with acetonitrile, and detected the solution at 425 nm by high-performance liquid chromatography.

##### 2.2.4.3. Determination of critical micelle concentration (CMC)

The amphiphilic block polymer DPC was composed of hydrophilic and hydrophobic ends. When DPC reached a certain concentration in the solution, it could self-assemble to form micelles. The minimum concentration that could form micelles in a solution was called the critical micelle concentration (CMC). The CMC of DPC was measured by pyrene fluorescence probe method.

##### 2.2.4.4. *In vitro* Cel release from DPC@Cel

The dialysis method was used to study the in vitro release test of DPC@Cel micelles profiled with or without MMP-2 enzyme. 1 ml of DPC@Cel was placed in a dialysis bag (3500 Daltons). The release media was 40 ml PBS pH 7.4 (containing 0.7% Tween 80). In the control group, we simulated the serum microenvironment and added 1.5/mL of MMP-2 enzyme to the release medium. The 50 ml centrifuge tube was placed in a 37 °C water bath shaker and shaken horizontally at a speed of 60 revolutions per minute. At a predetermined time node, 1 ml of release medium was collected and measured using HPLC. Each group was tested in parallel three times and the average value was taken.

#### 2.2.5. *In vitro* cellular uptake of DPC micelles

Raw 264.7 were seeded in a 12-well plate at a density of 2 × 10^4^ cells per well and cultured for 24 h, and pretreated with 100 ng/mL^−1^ lipopolysaccharide (LPS) for 24 h. After 4 hours, place 12-well plates containing different concentrations of DPC@coumarin-6 micelles or free coumarin-6 (80, 50, 25, and 10 ng/mL) under an inverted fluorescence microscope, and to observe the fluorescence intensity of coumarin-6.

After Raw 264.7 cells were fully attached and pretreated with 100 ng/mL^−1^ lipopolysaccharide (LPS) for 24 h, and disposed with DPC@Coumarin-6 micelles or free Coumarin-6 (Coumarin-6 concentration: 80 ng/mL). Then, we used an inverted fluorescence microscope to observe the fluorescence of Coumarin-6 in Raw264.7 cells cultured for 0.5, 1, 2, and 4 h.

To verify the targeting effect of DPC@Coumarin-6 on the type A scavenger receptor of activated Raw 264.7, we observed the cellular uptake incubated with micelles for 2 and 4 h in the with or without pre-activation of LPS.

In the control group, we added 1.5/mL of MMP-2 enzyme to the cell culture medium. After 4 hours, the inverted fluorescence microscope was used to compare the cell uptake with or without MMP-2 enzyme DPC@Cel.

#### 2.2.6. *In vitro* cell cytotoxicity assay

The cytotoxicity of free Cel, blank DPC, and DPC@Cel micelles were evaluated by the MTT experiment. Firstly, plant RAW264.7 cells (8 × 10^3^ cells/well) in a 96-well plate and culture for 24 h until they were fully attached. And then, LPS was added to the cells for 12 h. The original medium was discarded, and then 100 μL/well of fresh medium containing free Cel and DPC@Cel (the final concentrations of the Cel were 1000, 500, 250, 100, 50, and 10 ng/mL; the final concentrations of the blank DPC were 5000, 2500, 1000, 500, 250 and 100 ng/mL) were added and cultured for 24 or 48 h. Then, 20 μL MTT (5 mg/mL) was added to each well and kept in the dark place for 4 h. The absorbance of a 96-well plate at a wavelength of 570 nm was measured with a microplate reader.

#### 2.2.7. Anti-inflammation efficacy of Cel@Cel in vitro

Under the induction of LPS, macrophages were transformed into M1 macrophages, secreting a variety of cytokines. In this study, NO as pro-inflammatory cytokines, were determined to assess the inflammation levels. The Giress reagent method was used to detect the level of NO secreted by cells. First, logarithmic growth of RAW264.7 cells were collected and seeded in a 96-well plate and completely adhered to the wall. 100 μL of Cel and DPC@Cel of different concentrations were added to the 96-well plate, and the culture was continued for 2 hours. Then, LPS and cell suspension are mixed and cultured for 24 h. The cell supernatant is removed, after adding GiressA and GiressB reagents, the OD value is measured at the absorbance at 540 nm.

#### 2.2.8. Dpc@Cel induced apoptosis in RA-FLS

Apoptosis of RA-FLS was analyzed by cell necrosis and apoptosis kit. Since PI dye cannot penetrate the complete cell membrane, it can stain the nucleus of apoptotic and necrotic cells. In this experiment, PI and Hoechst 33342 staining were used to dye the nucleus, RA-FLS was inoculated and grown adherently into a 12-well plate, and different concentrations of free Cel and DPC@Cel were added to different wells. After 24 h, we observe the staining through an inverted fluorescence microscope.

#### 2.2.9. Animals

##### 2.2.9.1. Establishment and administration of rat model of rheumatoid arthritis

SD Rats (6–8 weeks old, 200 ± 25 g) were purchased from Jinan Pengyue Laboratory Animal Breeding Co. The FCA (Freund’s Complete Adjuvant) was injected subcutaneously into the right hind plantar of the rat (0.1 mL/head) to prepare an AIA (Adjuvant-induced rheumatoid arthritis) rat arthritis model. The rats in the control group were injected with the same dose of saline at the same site.

When the rats have secondary joint swelling, the joint swelling index score was performed, and the rats were randomly divided into groups according to the score. Next, these rats started continuous intravenous injections of free Cel, CPC@Cel and DPC@Cel micelles (1.5 mL/100g) for two weeks, and the model group was given the same dose of 0.9% sodium chloride solution.

A protocol for animal use was followed by the China Animal Protection Commission. All experiments on animals complied with ethical guidelines.

##### 2.2.9.2. *In vivo* real-time imaging

In this experiment, *in vivo* fluorescence imaging was used to evaluate joint targeting. We used DiR as a fluorescent probe and loaded it into different nanomaterials. Free DiR, CPC@DiR and DPC@Cel were intravenously injected into AIA rats, and the rats were photographed using the IVIS preclinical *in vivo* imaging system at 6, 12, and 24 h, respectively.

##### 2.2.9.3. Measurement of joint swelling rate

On the 14th day after inflammation, the secondary toe volume of the rat was measured with a toe volume meter. After the inflammation, the primary toe volume of the rat was measured every 3 days, and the joint swelling rate was calculated according to the formula.

##### 2.2.9.4. Arthritis index

After inflammation, the joint swelling score of AIA arthritis rats was performed.

##### 2.2.9.5. Weight determination

After inflammation and every two days after inflammation, the weight of the rats was measured and recorded.

##### 2.2.9.6. Histopathological analysis

After the last administration, 3 rats were selected from each group, major organs (heart, liver, spleen, lung and kidney) were collected, fixed in 4% paraformaldehyde, and paraffin embedded, then sectioned and stained with hematoxylin-eosin (H&E). Then, observation of pathological changes of rat tissues and organs by optical microscope

## 3. Results and discussion

### 3.1. Preparation and characterization of DPC

#### 3.1.1. Characterization of DPC

##### 3.1.1.1. H-NMR

**Figure 3.**
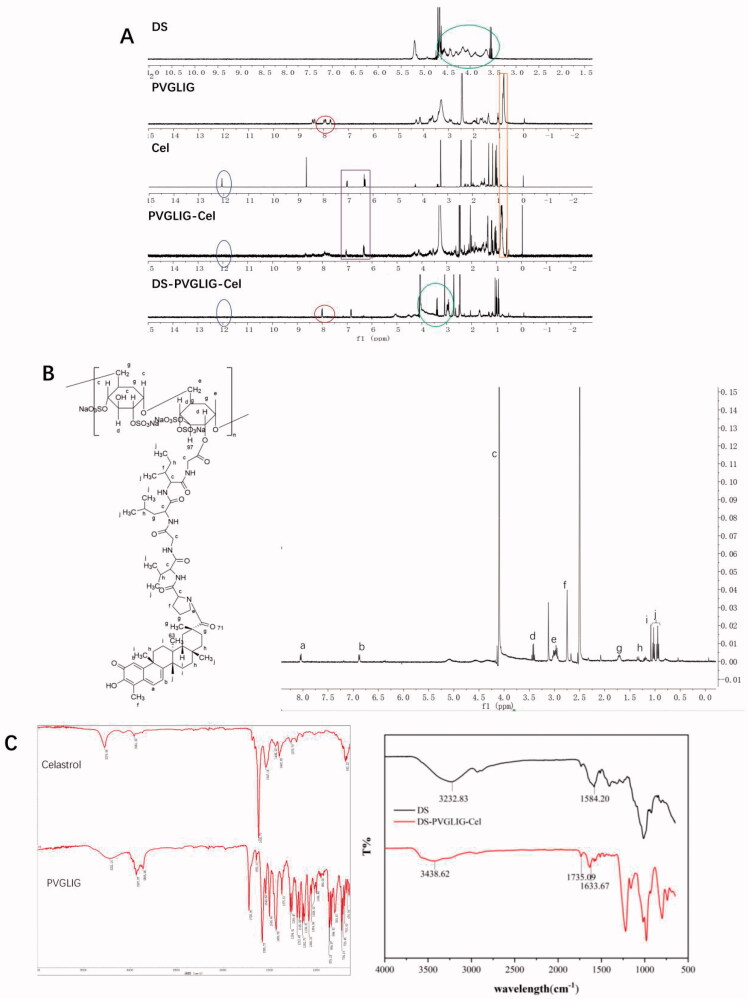
(A) 1H-NMR spectrum of DS, PVGLIG, Cel, PVGLIG-Cel and DS-PVGLIG-Cel. (B) 1H-NMR spectrum of DPC. (C) FT-IR spectrum of Cel, PVGLIG, DS and DPC.

The 1H-NMR spectra of DS, PVGLIG, Cel, PVGLIG-Cel and DPC are shown in the Figure 3(A). By observing the HNMR (Figure 3(B)), the chemical shifts at b (6.8 ppm) and a (7.2 ppm) was the double bond on six-membered ring peak of Cel. The chemical shifts at f (2.6 ppm) was the –CH_3_ of Cel. The chemical shifts at j (1.0 ppm) was the –CH_3_ of PVGLIG. The chemical shifts at c (4.0 ppm) was the –NH–CH_2_ of PVGLIG. The chemical shifts at d (3.4 ppm) was confirmed belonging to DS. In summary, the HNMR is basically consistent with DPC, and it can be judged that the structure is DS-PVGLIG-Cel.

##### 3.1.1.2. FTIR

The FTIR spectra of DS, PVGLIG, Cel, PVGLIG-Cel and DPC are shown in the Figure 3(C). It can be clearly seen that the peak (3000 − 3750 cm^−1^) where the hydroxyl group (–OH) is located is weakened, indicating that the hydroxyl group (–OH) is substituted. In the DPC spectrum, the tensile vibration of amide I (C = O peak) at 1633.67 cm^−1^ can be observed. In addition, a new tensile vibration band appeared at C = O at 1854.91 cm^−1^, which proved that DS has been successfully connected with PVGLIG. These results indicate that DPC has been successfully synthesized.

#### 3.1.2. Particle size, zeta potential, morphology, DS, EE% and stability

**Figure 4.**
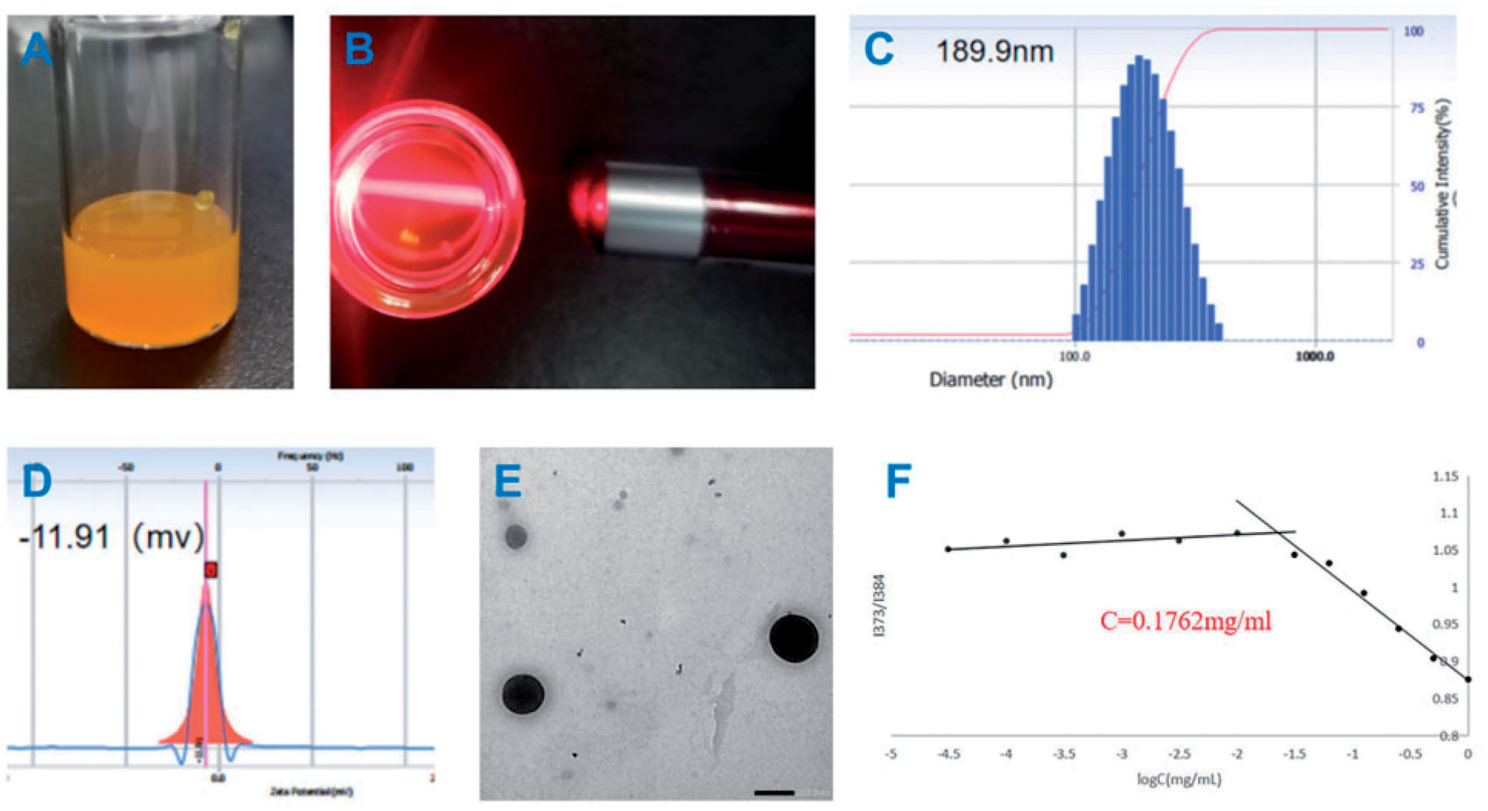
Appearance. (A) characterization of DPC@Cel micelles. (B) Tyndall effect of DPC@Cel in water, (C) particle size diagram, (D) and zeta potential, (E) the TEM picture, (F) critical micelle concentration of DPC.

The finished product after the drug-loaded micelles have passed through the 0.88 μm microporous membrane is shown in the Figure 4(A), the sample is orange and milky. When irradiated with a laser pointer, the sample can see obvious Tyndall effect (Figure 4(B)).The particle size, PDI and zeta potential were shown in Figure 4. The average size of DPC@Cel micelles was 189.9 nm (Figure 4(C)) and the PDI was 0.092, it shows that the particle size of the nanocarrier is narrow and uniform. The zeta potential of DPC@Cel micelles was −11.91 mV (Figure 4(D)). The EE% and DL% of DPC@Cel are 38.07 and 3.46%, respectively. TEM images (Figure 4(E)) showed that the nanoparticles were uniformly distributed spherical. As Table 1 showed, after 48 h, the particle size has not changed significantly, and the finished product has no precipitation or coalescence. In summary, DPC@Cel has a small particle size and good stability in blood circulation.

**Table 1.** The stability of DPC@Cel micelles.

Time (h)	PBS	PBS ± 20%FBS
2	193.8 ± 2.5	198.1 ± 3.6
12	206.1 ± 3.6	206.4 ± 3.3
24	209.0 ± 1.8	217.3 ± 2.9
48	219.8 ± 2.0	225.8 ± 3.8
72	229.8 ± 4.1	237.0 ± 5.0

#### 3.1.3. Critical micelle concentration of DPC

As Figure 4(F) showed, by calculation, the critical micelle concentration of DPC is 0.1762 mg/mL.

### 3.2. *In vitro* MMP-2-sensitive Cel release kinetics

**Figure 5.**
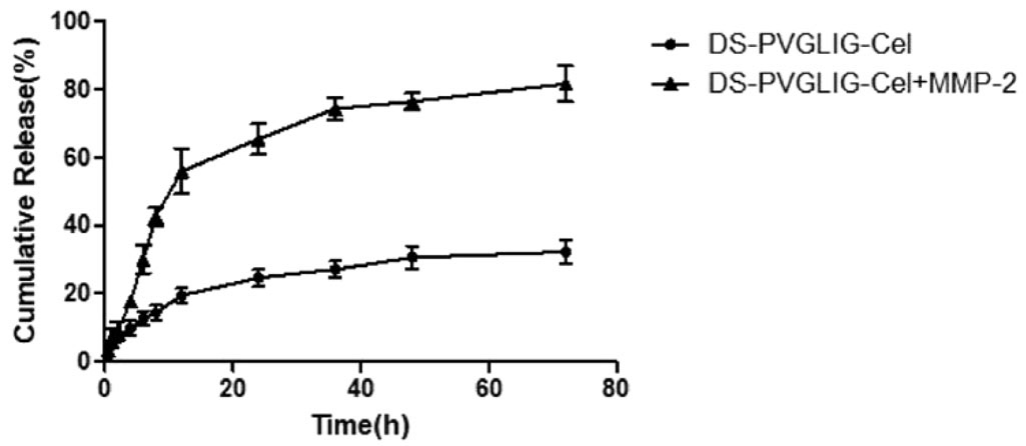
*In vitro* drug release of DPC@Cel incubated with or without the MMP-2 enzyme. Data are presented as mean ± SD (*n* = 3).

The *in vitro* MMP-2 enzyme-sensitive Cel release kinetics results were shown in Figure 5, which simulated the microenvironment of RA with high MMP-2 enzyme expression. The PBS solution with or without MMP-2 enzyme was used as the control release medium. As shown in Figure 5, DPC@Cel has good sensitivity to the MMP-2 enzyme, which was due to the characteristic that PVGLIG was hydrolyzed and broken by the MMP-2 enzyme. After 72 hours, 78% of Cel was released after incubation in the release medium containing the MMP-2 enzyme. By comparison, in the release medium that did not contain the MMP-2 enzyme, only about 30% of Cel was released. The *in vitro* release results showed that DPC@Cel had good MMP-2 enzyme selectivity and could specifically release Cel in inflammatory joints with high expression of MMP-2 enzyme.

**Figure 6.**
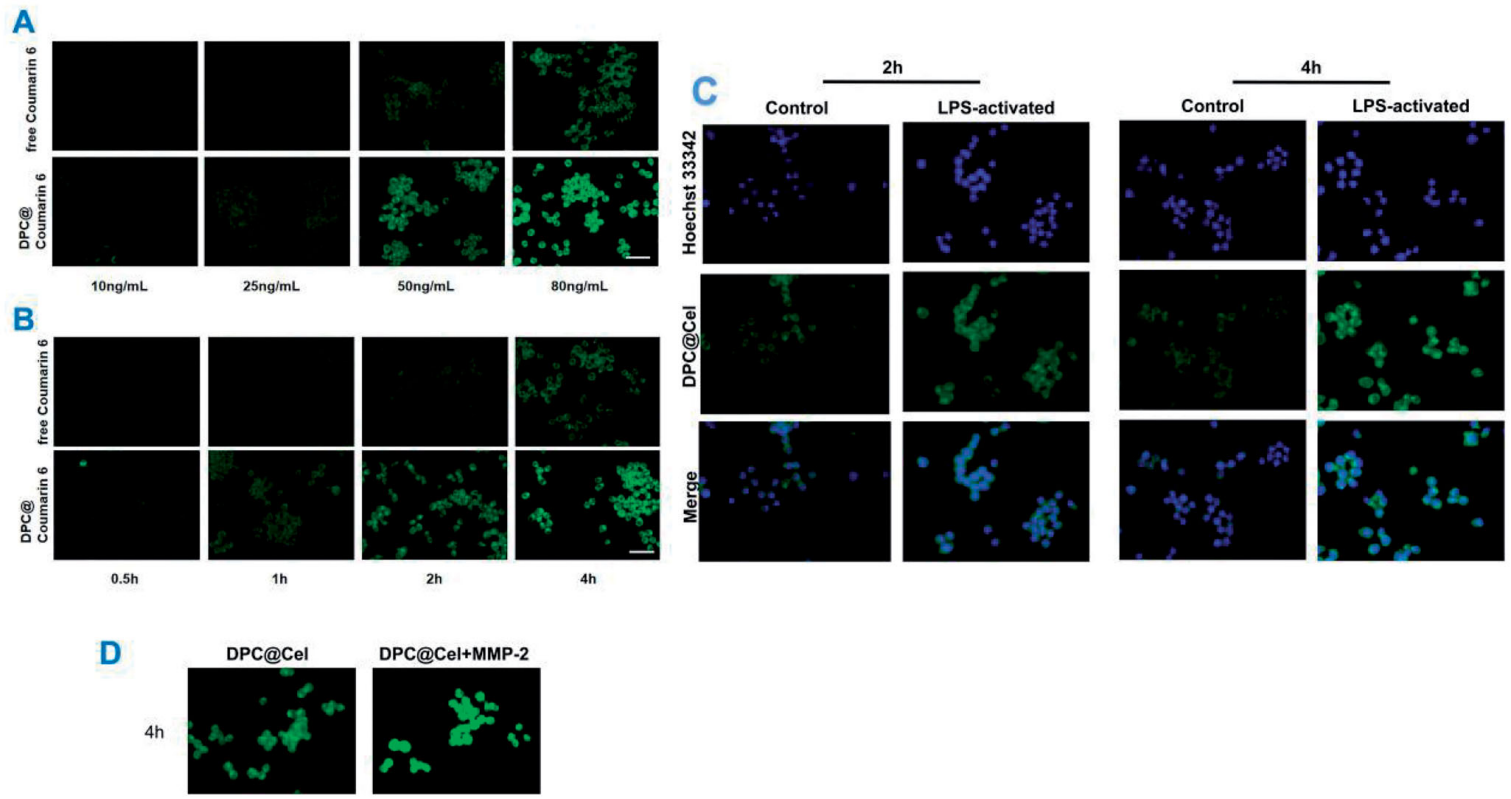
*In vitro* cellular uptake of DPC@Cel micelles in RAW264.7 cells. (A) The result of concentration dependence study. (B) The result of time dependence study. (C) Representative fluorescence images of RAW264.7 with or without LPS pretreatment which were incubated with free Cel or DPC for 2 and 4 h, respectively. Scale bar = 200 μm. (D) The result of the cellular uptake incubated with DPC@Cel micelles for 4 h in the presence and absence of MMP-2.

### 3.3. *In vitro* cellular uptake of DPC@cel micelles

Coumarin-6 has a strong fluorescence signal, so coumarin-6 is used as a fluorescence tracer. With the increase of the concentration of different preparations, the fluorescence intensity of the cells will increase, which indicates that the RAW264.7 cells have a concentration-dependent uptake behavior of free Cel and DPC@Cel (Figure 6(A)). Similarly, RAW264.7 cells were time-dependent on the cellular uptake behavior of free Cel and DPC@Cel (Figure 6(B)). The higher uptake proves that the nanocarrier micelles have better cell uptake efficiency.

Macrophages are considered to play key roles in RA. We used Hoechst 33342 to stain the nucleus of RAW264.7, it could be seen that the cytoplasm exhibits green fluorescence and the nucleus exhibits blue fluorescence (Figure 6(C)). As shown in the Figure 6(C), at 4 h, DPC@Cel was internalized by macrophages. In addition, compared with activated RAW264.7 cells, activated RAW264.7 cells shown stronger fluorescence, which indicated that DPC@Cel could effectively target activated macrophages, and quickly decomposed and released drugs.

When activated macrophages were incubated with excess MMP-2 enzyme early, the fluorescence signal of DPC@Cel micelles was significantly enhanced. This shows that MMP-2 enzyme can rupture DPC and accelerate the release process. In summary, DPC is a highly effective drug delivery system targeting activated macrophages (Figure 6(D)).

### 3.4. *In vitro* cell cytotoxicity study

**Figure 7.**
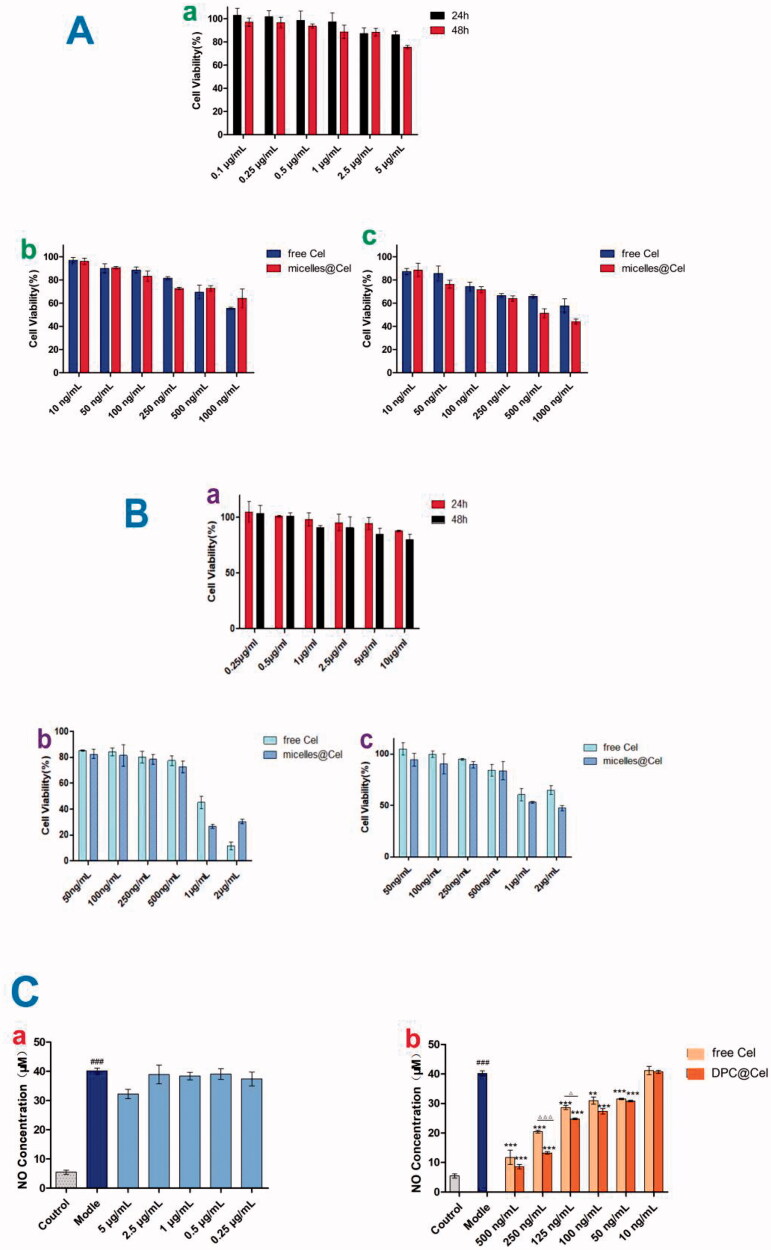
(A) (a) The cell viability of DPC at 48 and 24 h in Raw264.7 cells. (b, c) *In vitro* cytotoxicity of free Cel and DPC@Cel in RAW264.7 cells after treatment for 24 and 48 h. (B) (a) The cell viability of DPC at 48 and 24 h in RA-FLSs cells. (b, c) *In vitro* cytotoxicity of free Cel and DPC@Cel in RA-FLSs cells after treatment for 24 and 48 h. Data are showed as the mean ± SD (*n* = 3); *p < 0.05. (C) (a, b) The changes of renal cytokine responses, including NO, in RAW264.7 treated with DPC@Cel, free Cel. Data are presented as mean ± SD (*n* = 4), *p < 0.05; **p < 0.01.

As shown in Figure 7(A), the cytotoxicity of free Cel, blank DPC and DPC@Cel on RAW264.7 cells was investigated by using MTT assay. After 48 hours of culture, when the blank DPC micelle concentration was 5 μg/mL, the cell survival rate was still greater than 70%, which proves that blank DPC had low cytotoxicity. Within the concentration range, the cytotoxicity of blank DPC could be ignored. As shown in Figure 7(A), it is obvious that at the same concentration, DPC@Cel has higher cytotoxicity than free Cel. This is because DPC@Cel can be better internalized by RAW264.7 cells, which improves the bioavailability of triptorubin and therefore has higher cytotoxicity. At the same time, the results indicated that experimental concentrations of DPC@Cel from 10 to 250 ng/mL were safe for RAW264.7 cells.

RA-FLS was exposed to different concentrations of celastrol, blank DPC, DPC@Cel (2000, 1000, 500, 250, 100, 50 ng/mL) for 24 and 48 h. It was obvious that at the same concentration, DPC@Cel had higher cytotoxicity than free Cel. This is because DPC@Cel can better promote the apoptosis of RA-FLSs, which increases cytotoxicity. (Figure 7(B)) The results showed that in the concentration range of 100 ∼ 500 ng/mL, incubation of DPC@Cel for 24 or 48 h had no significant effect on cell viability.

As shown in the Figure 7(C), compared with the model group, both the Cel and DPC@Cel groups significantly reduced NO levels. In addition, compared with the Cel group, DPC@Cel has a better effect of inhibiting NO secretion. This is because DPC@Cel can be better internalized by macrophages, thereby increasing the bioavailability of Cel.

### 3.5. Cellular apoptosis and necrosis assay

**Figure 8.**
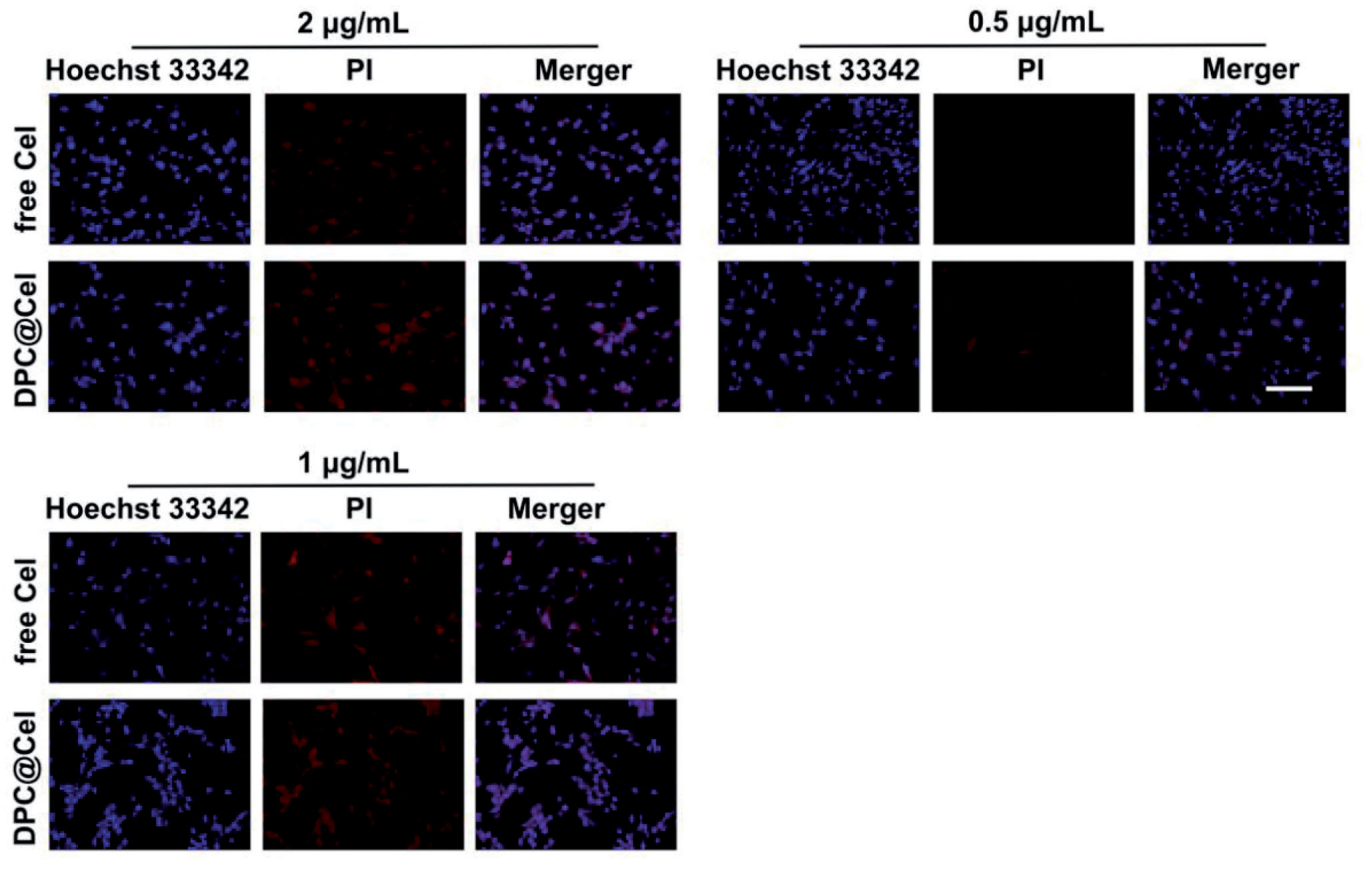
Cellular apoptosis and necrosis were examined by the PI and Hoechst 33342 staining assay (scale bar = 200 μm).

The results of the RA-FLS apoptosis and necrosis assay were shown in Figure 8. As the concentration of Cel increases, the red fluorescence intensity of RA-FLS becomes stronger. Especially when the concentration is 2 μg/ml, the number of RA-FLS cell apoptosis and necrosis increases. At the same time, under the same Cel concentration, DPC@Cel showed better ability to induce apoptosis and necrosis.

### 3.6. *In vivo* evaluation in the treatment of RA

#### 3.6.1. The effect of DPC@cel on the joint swelling rate of AIA rats

**Figure 9.**
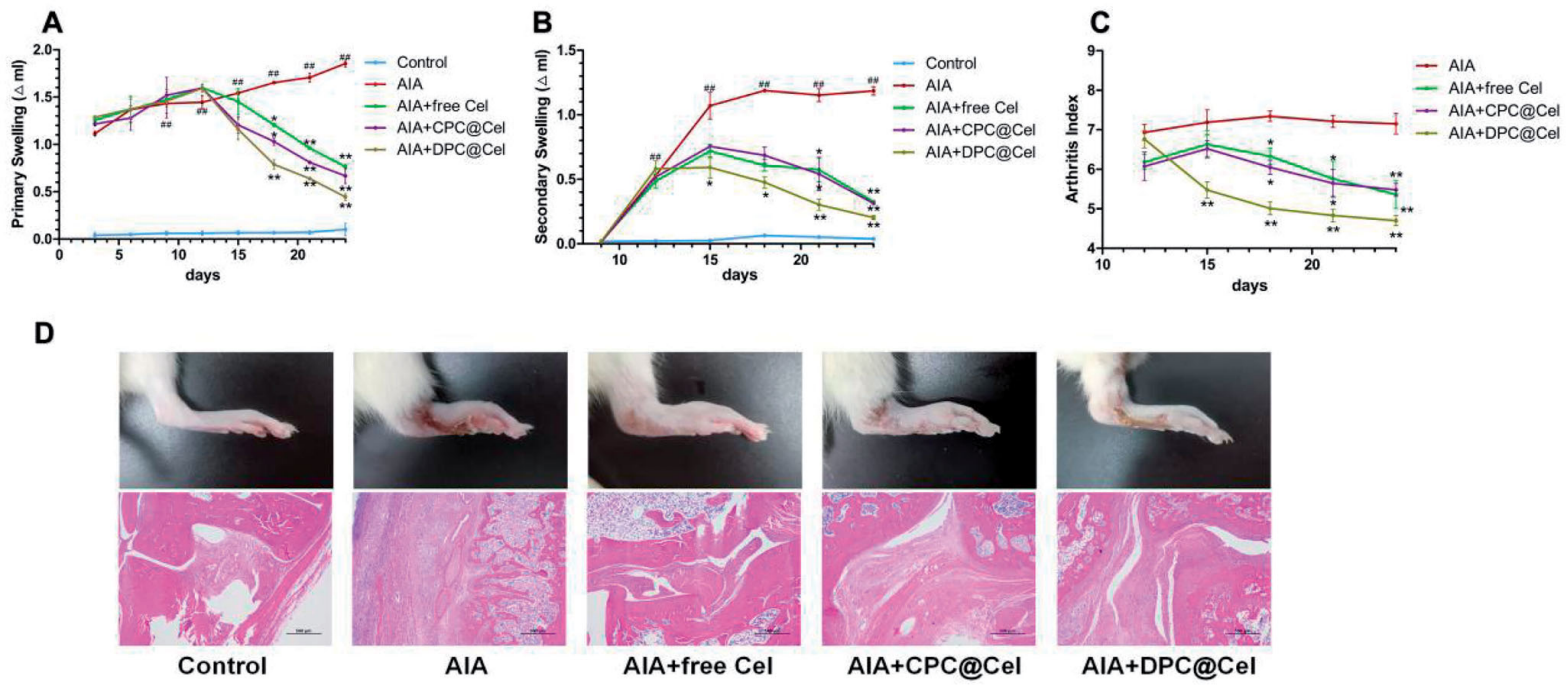
AIA clinical symptoms are effectively attenuated by administration of DPC@Cel. (A) The joint swelling rate of the right hind paw of rats were evaluated every 3 days until sacrifice. (B) The joint swelling rate of the left hind paw of rats were evaluated every 3 days until sacrifice. (C) The clinical scores of the right hind paw of rats were evaluated every 3 days until sacrifice. (D) Representative gross lesions, pathological staining with H&E. Data are presented as mean ± SD (*n* = 5), *p < 0.05, **p < 0.01, ***p < 0.001.

As shown in Figure 9(A,B), compared with the control group, the rat joint swelling rate in the model group was significantly increased (*p* < 0.01). Compared with the model group, free Cel, CPC@Cel and DPC@Cel could significantly inhibit the rat joint swelling rate. Compared with free Cel, DPC@Cel micelles group could significantly inhibit the swelling rate of rat joints. The results showed that DPC@Cel micelles group had better therapeutic effect than the free Cel in the treatment of RA.

#### 3.6.2. The effect of DPC@cel on the joint swelling index of AIA rats

As shown in Figure 9(C), the joint swelling index of rats in the model group was significantly increased (*p*＜0.01). Compared with the free Cel group, the DPC@Cel micelles group could significantly reduce the arthritis score of AIA rats.

#### 3.6.3. The histopathologic examination of joints

After the 12th day of treatment, the hindfoot swelling of SD rats in the DPC@Cel treatment group was significantly reduced, and inflammation was effectively controlled. In the histopathological examination of joints, DPC@Cel-treated mice had significantly reduced synovial inflammation and bone erosion compared with untreated and CPC@Cel-treated SD rats (Figure 9(D)), indicating that the treatment of rheumatoid arthritis was successful.

#### 3.6.4. *In vivo* real-time imaging

**Figure 10.**
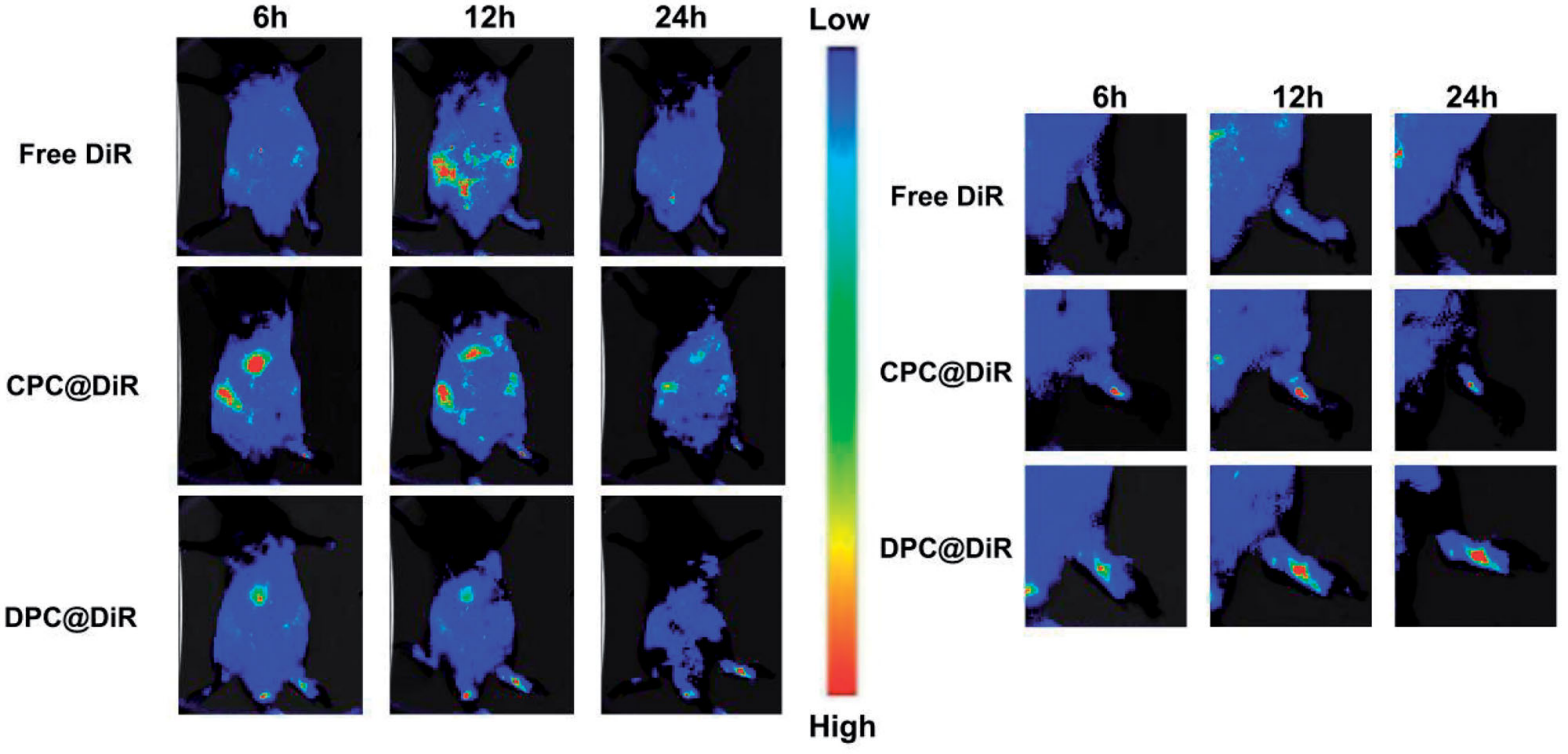
The *in vivo* fluorescence images of free DiR, CPC@DiR, and DPC@DiR.

It can be seen from the Figure 10 that the fluorescence intensity in the inflamed joints of the DPC@DiR-treated group is more obvious than that of the free DiR-group and CPC@DiR-treated group. The free DiR-treated group has almost no fluorescence accumulation in the inflamed joints. In addition, the DPC@DiR-treated group stayed longer in the joints. The experimental results show that DPC has good targeting and sustained-release effect. Both the free DiR-treated group and CPC@DiR-treated groups showed a strong systemic distribution, which indicates that DPC@DiR-treated has lower systemic toxicity.

#### 3.6.5. Preliminary evaluation of *in vivo* safety

##### 3.6.5.1. The effect of DPC@Cel on rat body weight

**Figure 11.**
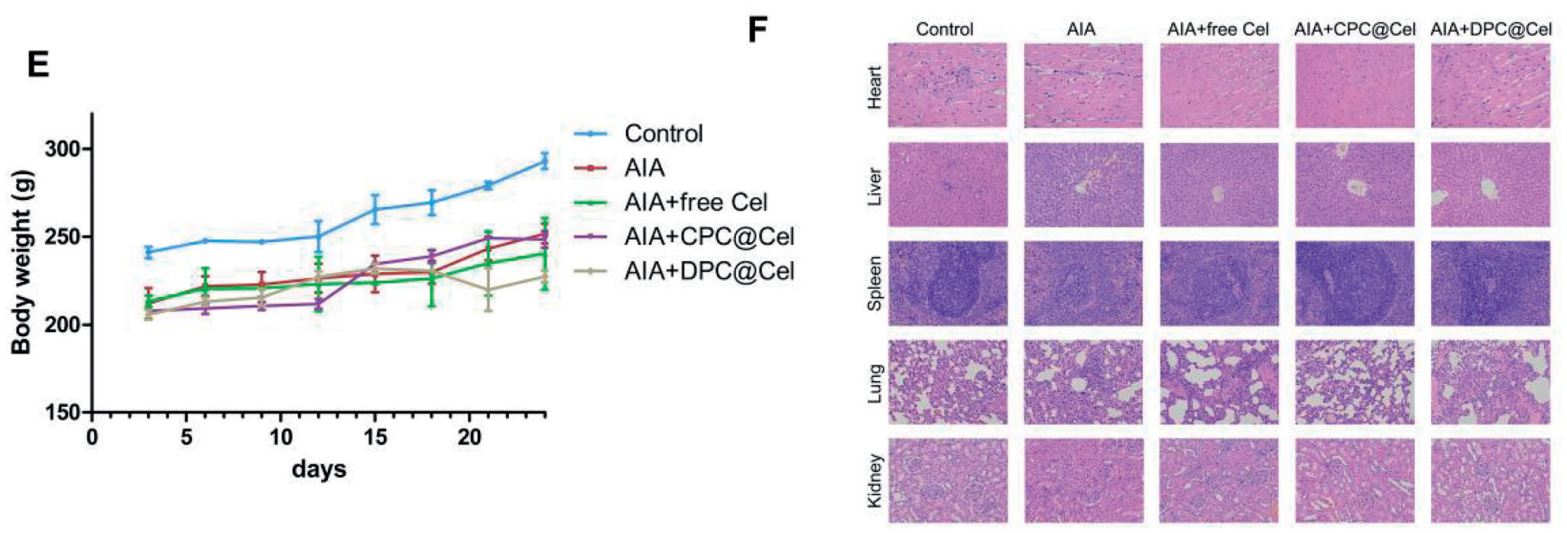
Preliminary evaluation of *in vivo* safety: (A) Body weight changes of different Cel preparations. (B) Histological changes of major organs after administration.

As shown in Figure 11(E). Compared with the control group, the weight of the rats in the model group was significantly reduced, and the weight of the rats in the DPC@Cel micelles group was not significantly reduced. The results show that DPC@Cel has little effect on the weight of the adjuvant arthritis rats.

In addition, we observed that the main organs of SD rats in different groups stained by H&E had no obvious histological damage. Therefore, it is proved that DPC@Cel has no significant systemic toxicity to SD rats (Figure 11).

## 4. Conclusions

In summary, we successfully prepared an amphiphilic nanocarrier with MMP-2 enzyme sensitivity based on dextran sulfate targeting, and characterized it by various methods. Furthermore, in vitro study certified that DPC@Cel can release drugs specifically in the inflammation joints. The sensitive peptides of MMP-2 were used to release drugs specifically at the site of inflammation, which provided new ideas in the treatment of RA. The cellular cytotoxicity and uptake analysis indicated that DPC@Cel increases the accumulation of celastrol in RAW264.7 cells, and improves the release rate of the drug in the process of inflammatory joints.

Compared with DPC@Cel, the prepared Cel showed enhanced cytotoxicity. In addition, DPC@Cel could promote the apoptosis of RA-FLS cells in a concentration-dependent manner. Compared with Cel group, DPC@Cel had a higher inhibition rate of cell inflammatory factors. The results of in vivo experiments shown that DPC@Cel had a better therapeutic effect. In addition, the results of in vitro safety evaluation experiments proved that DPC@Cel has insignificant systemic toxicity. In a word, a MMP-2 enzyme-sensitive and precise RAW264.7 cell targeting nanoparticle containing Cel was successfully prepared, which had broad application prospects in the treatment of rheumatoid arthritis in the future.
